# Detection of Selection Signatures and Genome-Wide Association Analysis of Body Weight Traits in Xianan Cattle

**DOI:** 10.3390/genes16060682

**Published:** 2025-05-30

**Authors:** Huaini Zhu, Xiaofeng Li, Man Zhang, Siyu Liu, Yan Zhang, Ying Zheng, Zhitong Wei, Mingpeng Han, Hetian Huang, Tong Fu, Dong Liang

**Affiliations:** 1 College of Animal Science and Technology, Henan Agricultural University, Zhengzhou 450046, China; hnzhu7799@163.com (H.Z.); 18338210615@139.com (M.Z.); 16650768076@163.com (S.L.); zhangy20210000@163.com (Y.Z.); zhnny9197805105@yeah.net (Y.Z.); futong2004@126.com (T.F.); 2 College of Veterinary Medicine, Henan Agricultural University, Zhengzhou 450046, China; nytndlxf@163.com; 3 Henan Provincial Seed Industry Development Center, Zhengzhou 450046, China; mingpeng999@163.com

**Keywords:** Xianan cattle, GWAS, body weight, MANEA gene

## Abstract

**Background:** Xianan cattle, the first cross-bred beef cattle developed in China, are recognized for their rapid growth, tolerance to rough feed, and high meat yield. These characteristics make them a valuable model for studies aimed at improving beef production traits. **Methods:** In this study, two complementary gene mapping strategies, selection signature analysis and association analysis, were employed to identify candidate genes associated with body weight. The analyses utilized resequencing data comprising 16,250,950 high-quality single nucleotide polymorphisms (SNPs). Twenty independent variables showed significant correlations with body weight, with effect sizes ranging from 239 kg to 629.37 kg, while controlling for a false discovery rate (FDR) of less than 0.5. **Results:** The most prominent signal was identified in the 54.24–54.39 MB region on chromosome 9, which contains the MANEA gene. Furthermore, we investigated the functional role of the MANEA gene at the cellular level. siRNA-mediated knockdown of MANEA resulted in significant alterations in the expression of downstream genes, notably MGAT1, MGAT3, FUT8, and HK1. Among these, the expression of MGAT1 was markedly increased, showing an increase of up to 600-fold compared to the control. **Conclusions:** These results offer critical insights into the molecular mechanisms underlying body weight regulation and provide a foundation for developing strategies to enhance economically important production traits in beef cattle.

## 1. Introduction

The rapid development of beef cattle production not only mitigates competition for grain resources between humans and livestock but also contributes significantly to improving human dietary structures [1]. Additionally, beef cattle farming plays a vital role in advancing China’s rural revitalization initiatives, driving economic growth in rural areas, and supporting agricultural sustainability [2]. In response to these demands, China has substantially increased its investment in beef cattle farming and related research over recent decades [3]. As one of the world’s largest producers and consumers of beef, ensuring a stable and sustainable supply of beef products is crucial for China. Achieving this goal depends on the establishment of a scientific and comprehensive breeding system [4]. To unlock the full production potential of native cattle breeds, breeders have actively introduced foreign bloodlines through hybridization, resulting in new breeds that are better adapted to local environmental conditions [5] and production requirements. Xianan cattle, a hybrid of Charolais and Nanyang cattle, represent the first specialized beef breed developed in China. This breed exhibits several desirable traits, including early maturity, rapid growth, high-quality meat, and low dystocia rates [6]. These characteristics not only enhance production efficiency but also increase profitability for farmers, making Xianan cattle a significant contributor to the sustainable development of China’s beef industry.

The body weight (BW) trait is a key trait in beef cattle breeding, as it directly influences several critical aspects of cattle production, including growth rate [7], feed efficiency, carcass yield [8], dystocia rates [9], and overall profitability. Genetic selection for body weight not only supports economic objectives but also contributes to broader efforts aimed at enhancing sustainability and efficiency in the beef industry. Understanding the genetic basis of body weight is therefore essential for optimizing breeding strategies to meet both economic and production goals. Recent advances in molecular biology, genetics, and bioinformatics have significantly accelerated research in animal genomics. Among these advancements, genome-wide association studies (GWAS) have proven invaluable in identifying the genetic architecture underlying complex traits, such as body weight, which are often challenging to measure directly in cattle. In cattle populations, GWASs have been extensively applied to identify loci associated with body weight, thereby improving our understanding of the genetic mechanisms driving growth traits. For example, Igoshin et al. [10] used the GGP HD150K array to identify the CCND2 gene, which has been associated with average daily weight gain and body mass index in Siberian cattle. Similarly, Yeo et al. [11] reported that RPGRIP1L and IRX3 influence the function of FTO, a gene with a significant role in regulating growth and energy metabolism. Additionally, Zhang et al. [12] identified MAPK3, LDB2, and LRP1B as key genes contributing to muscle growth and development in Inner Mongolia Cashmere goats.

However, comprehensive studies on the body weight traits of indigenous Chinese cattle remain limited. After years of selective breeding, Xianan cattle have developed significantly larger body weights compared to other native cattle breeds, making them an ideal model for investigating the genomic basis of complex traits [13]. The aim of this study was to identify candidate genes associated with body weight in Xianan cattle using a genome-wide association study (GWAS). This genetic analysis seeks to elucidate the molecular basis of body weight variation, a critical trait affecting productivity in livestock. The findings may subsequently inform strategies for genetic improvement programs targeting enhanced growth and efficiency in this breed.

## 2. Materials and Methods

### 2.1. Ethics Statement

The animal study protocol was approved by the Ethics Committee of Henan Agricultural University (protocol code HNND2025031323; approval date: 10 March 2025).

### 2.2. Sample Collection and Phenotypic Measurements

A total of 149 healthy adult female Xianan cattle from the Xianan Cattle Farm in Biyang County, Zhumadian City, Henan Province, were used in this study. Weight phenotype data were recorded using an electronic weighing scale. Approximately 40 mL of blood was collected from the jugular vein by a veterinarian, using ACD (acid–citrate–dextrose) as the anticoagulant. The blood samples were collected in vacuum tubes and stored at −80 °C for subsequent analysis.

### 2.3. Resequencing Data and Variant Discovery

Genomic DNA was extracted from the blood samples of Xianan cattle using the Tissue Genome DNA Extraction Kit (DC112) from Nanjing Nuoweizan Biotechnology. The determination of genomic DNA integrity was carried out using gel electrophoresis and NanoDrop spectrophotometry. We established a double-ended sequencing library using the DNBSEQ-T7 platform, with a size of 15 bp. Trimmomatic software (v0.39), which serves as a flexible trimmer for processing Illumina sequencing data by removing low-quality bases and trimming adapter sequences, was used to filter the generated FASTQ data, resulting in clean reads [14]. The BWA-MEM algorithm with default parameters [15] was then used to align these clean reads with the ARS-UCD1.3 reference genome. PCR duplicates were removed using the MarkDuplicates module of the Picard toolkit (https://broadinstitute.github.io/picard/index.html) (URL, accessed on 5 March 2023), and indels were realigned using the IndelRealigner module of GATK (v3.8) [16]. To ensure base quality, recalibration was carried out using the BaseRecalibrator module of GATK. Variant calling was conducted using the UnifiedGenotyper module of GATK, followed by post-detection filtering with the following parameters: QD < 2.0, FS > 60.0, MQ < 40.0, MQRankSum < −12.5, ReadPosRankSum < −8.0, AF < 0.01, DP < 800. SNPs with minor allele frequencies < 0.05 and those deviating from Hardy–Weinberg equilibrium (*p* < 10^−6^) were refused, as well as individuals with >10% missing genotypes. To ensure high accuracy in variant calling, PLINK software v1.90 was used to remove duplicates and filter non-biallelic SNPs. After quality control, genotyping was performed using Beagle 5.4 software with default parameters [17].

### 2.4. GWAS

A genome-wide association study (GWAS) was conducted using the Fixed and Random Model Circulation Probability Unification (Farm-CPU) approach [18]. It merges fixed-effect models (FEM) and random-effect models (REM) iteratively. SNP genotypes were encoded as 0, 1, and 2 using Plink software (v1.90) [19]. SNPs exceeding the threshold in FEM were identified as pseudo-quantitative trait nucleotides (QTNs). These pseudo-QTNs were further validated using REM, with kinships constructed from alternative sets of pseudo-QTNs. FEM and REM were run alternately until no new significant pseudo-QTNs were identified.

The FarmCPU model alternated between fixed effects and random effects to improve the site detection rate, using pseudo QTNs as covariates. The fixed-effects model was as follows:

$$y_{i}=M_{i1}b_{1}+M_{i2}b_{2}+\cdots +M_{it}b_{t}+S_{ij}d_{j}+e_{i}$$

where $y_{i}$ denotes the observed vector of the trait i in the individual animals. The genotypes of the associated loci are represented by $M_{i1},M_{i2},\ldots ,M_{it}$, where t corresponds to the number of loci under consideration. The effect values for each associated locus are denoted as $b_{1},b_{2},\ldots ,b_{t}$. The term $S_{ij}$ refers to the genotype of the j-th genetic marker for the i-th individual, with $d_{j}$ representing the corresponding effect of this marker. Finally, $e_{i}$ represents the random residual for the i-th individual, which follows a normal distribution with a mean of 0 and a variance $\sigma_{e}^{2}$. The random effects model is structured as follows:

$$y_{i}=u_{i}+e_{i}$$

where $y_{i}$ is the observed vector for the trait of the i-th individual, $u_{i}$ is the total genetic effect of the i-th individual, and $e_{i}$ is the random residual vector, which follows a normal distribution with mean 0 and variance $\sigma_{e}^{2}$.

To account for potential false positives, the first three principal components from the PCA were included in the FarmCPU model.

### 2.5. Selective Sweep Analysis

To investigate selective sweeps, the samples were ranked according to weight, from the lightest to the heaviest. The top 30 lightest and top 30 heaviest individuals were selected as two distinct groups for population differentiation analysis using the fixation index (FST). FST is a critical metric for quantifying genetic differentiation between populations, with higher FST values indicating greater differentiation between groups. In this study, FST values were calculated using VCFtools software (v0.1.16) [20].

### 2.6. Gene Function Annotation

All significant SNPs were annotated using SnpEff v4.3t [21], with gene annotations derived from the reference genome ARS-UCD1.3. Based on this genome annotation, SNPs were classified into several categories: exonic regions, 5′ and 3′ untranslated regions (UTRs), intronic regions, splice sites (within 2 bp of a splice junction), upstream and downstream regions (within 1 kb of the transcription start site), and intergenic regions.

### 2.7. Cell siRNA Interference Experiment

In this experiment, siRNA oligonucleotide fragments were obtained from Ruibo Company. The order included five siRNAs, each in separate tubes: three different siRNAs designed for the same target gene, one positive control (Actin), and one negative control (NC).

The experimental procedure was carried out as follows: Bovine fibroblast cells, in good condition, were seeded in 6-well culture plates at a density of approximately 60%, with 2000 µL of complete medium per well, ensuring 80% confluence at the time of transfection. After transfection, the medium was refreshed 4 ~ 6 h later. To ensure the continued suppression of target gene expression, siRNA transfection was repeated on day 4 of differentiation to maintain low-level expressions of the target genes. Total RNA was extracted using TRIzol reagent, followed by chloroform extraction and isopropyl alcohol precipitation. The RNA was then dissolved in RNase-free water. One microgram of RNA was used for cDNA synthesis in a 20-μL reaction. Reverse transcription was performed, and quantitative PCR (RT-qPCR) was carried out using ChamQ Universal SYBR qPCR Master Mix (Q711, Vazyme), with 1 µL of cDNA and 0.2 µM primers (Table A1). The PCR conditions were as follows: initial denaturation at 95 °C for 30 s, followed by 40 cycles of denaturation at 95 °C for 10 s, annealing at 60 °C for 30 s, and a final melting curve step, consisting of 95 °C for 15 s, 60 °C for 60 s, and 95 °C for 15 s. Differentiation levels were assessed using fluorescence microscopy and RT-qPCR analysis. The expression levels of target genes were compared to those of the negative control and marker genes using GraphPad Prism 5, with statistical significance determined by a *t*-test. Expression data were visualized and compared. To account for variations in RNA content and differences in sample processing, the expression of the target gene was normalized against the reference gene 18S rRNA using the ΔΔCt method.

### 2.8. Statistical Analysis

The weight data of Xianan cattle were statistically analyzed. It was found that the data followed a normal distribution. Descriptive statistics, including mean, standard deviation (SD), median, and interquartile range (IQR) were calculated. Box plots were generated for the 30 heaviest individuals, the 30 lightest individuals, and the entire study population. The first quartile (Q1) and the third quartile (Q3) were used to determine the IQR, which represents the central 50% of the data. Outliers were identified as values that exceeded the whiskers by 1.5× IQR. These statistical measures provide a comprehensive overview of the weight distribution of the studied population.

## 3. Results

### 3.1. Descriptive Statistics

**Figure 1:**
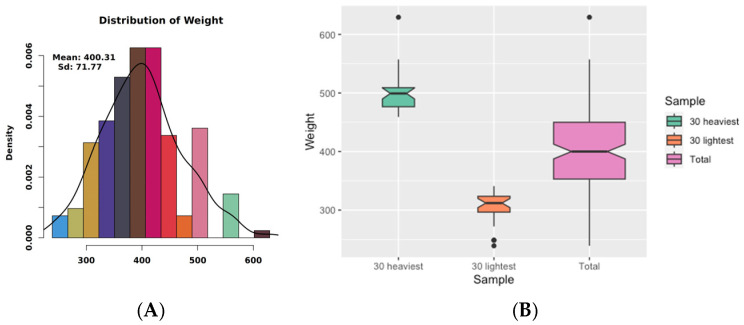
Normal distribution and boxplot analysis of body weight in Xianan cattle. (**A**) Normal distribution of body weight. (**B**) In the boxplot, the line in the middle of each box represents the median of the data. The top and bottom of the box are the top quartile (Q3) and the bottom quartile (Q1) of the data.

Based on the phenotypic data (Table 1), the body weight statistics obtained in this study are normally distributed (Figure 1A). The coefficient of variation (CV) for body weight (BW) in the dataset is 17.78%. Body weight distributions were compared across three cohorts: the 30 heaviest individuals, the 30 lightest individuals, and the full study population (Figure 1B). The median weights for these groups were 499.17 kg, 312 kg, and 400 kg, respectively. The boxes span the first (Q1) to third quartiles (Q3), representing the interquartile range (IQR) that contains the central 50% of measurements. Outliers, defined as values exceeding 1.5× IQR beyond the whiskers, included one extreme value in the heaviest cohort (629.31 kg), two in the lightest cohort (239 kg, 248.6 kg), and one in the full dataset.

### 3.2. Quality Control and Population Structure

**Figure 2:**
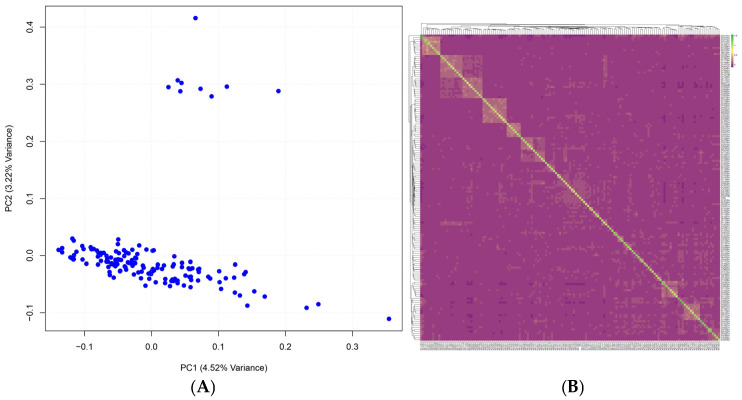
Relationships between individuals. (**A**) Population structure identified by principal components analysis, PC1 and PC2. (**B**) Genomic Relationships Matrix among Xianan individuals.

Whole-genome resequencing was conducted on 149 Xianan cattle, with an average sequencing depth of 16×. A total of 99.87% of the reads were successfully mapped to the reference genome, resulting in the identification of 16,287,842 SNPs. After quality control (QC), a total of 16,250,950 variants and 149 cattle were retained. To assess the population structure of Xianan cattle, we performed principal component analysis (PCA) and kinship analysis using the Genomic Relationship Matrix (G-matrix) based on the retained SNP data. PCA showed that most individuals are relatively concentrated, while only nine individuals exhibited stratification. Specifically, the first principal component (PC1) accounted for 4.52% of the total genetic variation, while the second principal component (PC2) explained 3.22%. Although a few individuals were identified as outliers, there was no obvious stratification within the population (Figure 2A). The results of the G-matrix analysis are shown in Figure 2B. The lighter the color, the more distant the relationship. Conversely, the darker the color, the closer the relationship. Lighter regions indicate moderate genetic relationships, while darker regions represent closer genetic relationships. From the G-matrix, it is apparent that most individuals are genetically distant from one another, with only a few exhibiting closer genetic relationships, which is consistent with the PCA results.

### 3.3. GWAS Result for Body Weight Trait

**Figure 3:**
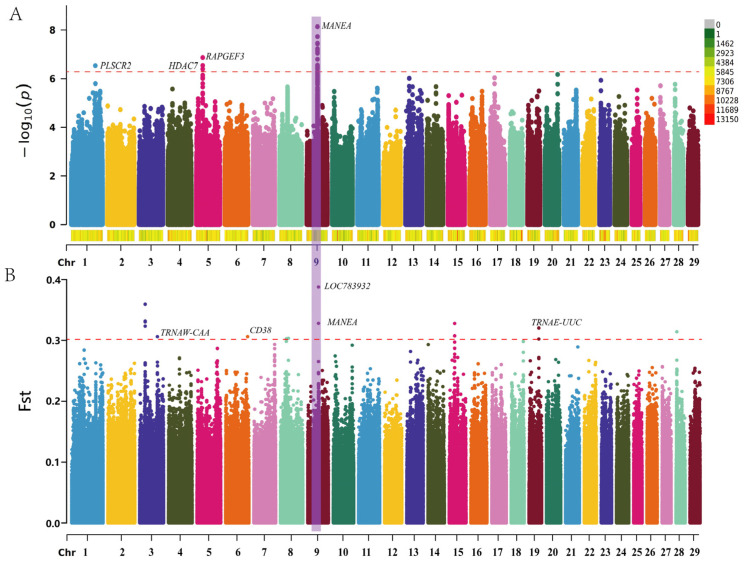
Manhattan plot of association results from genome-wide association analysis and F_ST_. (**A**) Manhattan plot of genome-wide association results for body weight traits using FarmCPU model analysis, highlighting genomic regions associated with body weight. The most significant SNPs were identified on chromosome 9. (**B**) Manhattan plot of F_ST_ values across the genome, identifying regions with significant genetic differentiation. The MANEA gene on chromosome 9 was found to be significant.

**Figure 4:**
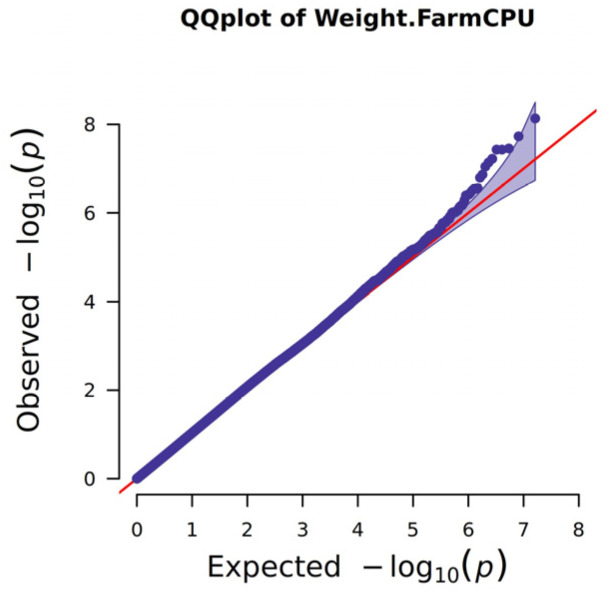
Quantile–quantile plot of GWAS for body weight traits in Xianan cattle. The red line (expected values) illustrates the null hypothesis of no true association. The observed values (purple dots) are a deviation from this line. The purple shaded area illustrates the confidence interval of the *p*-values.

Gene localization was investigated through genome-wide association studies (GWAS) and selective signal analysis. The GWAS results, with a false discovery rate (FDR) threshold of <0.5, identified a total of 20 significant loci, which were distributed across chromosomes 1, 5, and 9. These loci corresponded to the following genes: PLSCR2 (phospholipid scramblase 2), HDAC7 (histone deacetylase 7), RAPGEF3 (Rap guanine nucleotide exchange factor 3), and MANEA (α-endomannosidase). Notably, the majority of these loci were located on chromosome 9, with the MANEA gene being the most prominent (Figure 3A). Selective signal analysis identified 16 loci within the top 0.001% of SNPs, which were distributed across chromosomes 3,6, 9, and 19. These loci included TRNAW-CAA, CD38, LOC783932, MANEA, and TRNAE-UUC, with the MANEA gene on chromosome 9 being the most significant (Figure 3B, Table A2). Q-Q plot analysis indicated that the association model effectively controlled for population stratification, and significant sites were detected. In the Q-Q plot, the blue dots closely align with the red line, suggesting a strong fit of the model to the data (Figure 4). The most significant SNPs were located within the MANEA gene on chromosome 9. These SNPs were categorized into intronic variants, 5′untranslated region (UTR) variants, and intergenic regions, as shown in Table 2.

### 3.4. Cell siRNA Interference Experiment

**Figure 5:**
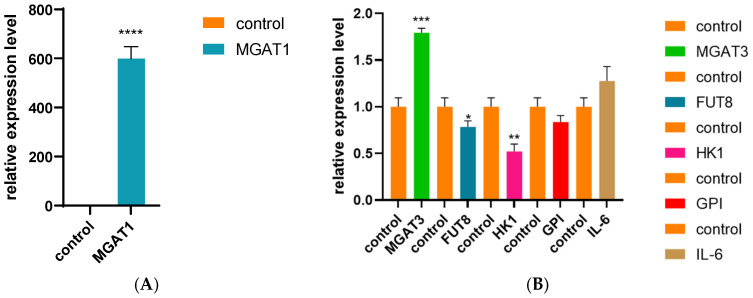
Expression of downstream genes of MANEA after siRNA interference. The significance level between the control group and each gene treatment was determined by *t*-test. * *p* < 0.05 was considered statistically significant. (**A**) Relative expression levels of MGAT1 in bovine fibroblasts after siRNA interference. (**B**) Relative expression levels of MGAT3, FUT8, HK1, GPI, and IL-6 after siRNA interference compared to the control group. ** *p* < 0.01, *** *p* < 0.001, **** *p* < 0.0001.

In order to further verify the function of the MANEA gene, we selected its downstream genes MGAT1, MGAT3, FUT8, HK1, GPI, and IL-6 for siRNA interference assays. After siRNA was successfully introduced into bovine fibroblasts, the target gene interference results were analyzed using fluorescence microscopy and RT-PCR, as shown in Figure 5. We found that the expression levels of MGAT1, MGAT3, and IL-6 were upregulated compared to the control group. A *t*-test showed that the upregulation of MGAT1 was significantly higher than that of the other genes, with an increase of up to 600-fold; however, the increase in IL-6 expression was not statistically significant. On the contrary, the expression levels of FUT8, HK1, and GPI genes were downregulated, with FUT8 and HK1 showing statistically significant reductions (Table A3).

## 4. Discussion

Beef plays a vital role in global food and nutrition security by providing high-quality protein and key trace elements that the body needs [22]. At present, Xianan cattle breeding is mainly based on purebred Xianan cattle, which produce tender and delicious meat with low fat content and are highly favored by consumers. As a beef cattle breed successfully cultivated in China, there is limited research on its production traits, and it is of great significance to study the weight traits of Xianan cattle to improve their economic traits and those of other breeds of beef cattle.

In this study, we found that the phenotypic data of 149 Xianan cattle followed a normal distribution, indicating that the group we selected was representative, with individuals with extreme body weight and statistical regularity. In addition, we analyzed population structure and combined the results of GWAS and selection signature analysis. We identified PLSCR2, RAPGEF3, HDAC7, MANEA, TRNAW-CAA, CD38, LOC783932, and TRNAE-UUC genes.

In this study, we found that MANEA was not only the most significant gene in GWAS but also the intersection gene of GAWS and FST. Through a literature search of MANEA, we found that most the research on MANEA focuses on the treatment of mental illness and cancer. For example, Jensen et al. performed multi-stage association analyses in European American and African American cohorts, starting with four psychiatric disorders, and found that a MANEA single nucleotide polymorphism (SNP; Rs1133503) is associated with anxiety disorders [23]. Yuanfa Feng et al. identified candidate genes, including MANEA, through single-cell RNA sequencing analysis while studying enzalutamide [24]. In a study of MANEA related to growth and development, Loic Yengo et al. [25] conducted a cross-racial GWAS analysis and identified 12,111 SNPs, which provided a comprehensive map of the vast majority of common height-associated variants. Two of these SNPs (rs13205436 and rs9481950) were located in the MANEA gene. MANEA’s downstream fat-related genes were MGAT1, MGAT3, FUT8, HK1, GPI, and IL-6. The most significant difference between the MGAT1 gene and the control group was observed in the bovine fibroblast siRNA interference assay. MGAT1 is a key enzyme involved in protein and lipid glycosylation. MGAT1 affects tumor progression and improves prognosis by regulating macrophage glycosylation levels. It plays an important role in pancreatic ductal adenocarcinoma. In addition, MGAT1 is a member of the monoacylglycerol acyltransferase family, and genetic variants downstream of MGAT1 have been reported by JA Jacobsson et al. as potentially influencing obesity susceptibility. In Qanbari’s research [26], the MGAT1 gene was reported to be related to muscle formation; Tapia-Rivera also pointed out that the MGAT1 gene plays a role in human obesity [27]. These findings are consistent with the conclusion of this study that the MGAT1 gene is highly correlated with body weight in cattle.

Despite revealing the important role of MGAT1 in body weight, our study has some limitations. The limited sample size and data heterogeneity may affect the generalizability and reliability of the results. Future studies should include larger sample sizes and integrate data from different sources for comprehensive analysis.

As a popular breed of beef cattle among consumers, improving the production performance of Xianan cattle is of great significance in meeting the needs of modern life. Against the backdrop of increasing demand for beef, the growth traits of Xianan cattle are a major research focus. Identifying and validating candidate genes related to body weight provides scientific and theoretical support for modern beef cattle breeding.

## 5. Conclusions

The increasing global demand for high-quality beef necessitates improved production efficiency in domestic cattle breeds. In this study, we identified MANEA as a critical modulator of growth traits in Xianan cattle, a breed distinguished by accelerated growth rates and premium meat characteristics. Functional analyses revealed that MANEA exerts its effects through the glycosylation-dependent regulation of metabolic pathways associated with nutrient utilization. This study found that significant SNP loci on the MANEA gene can be used for molecular marker-assisted selection to improve the accuracy of weight trait selection in cattle herds.

## Figures and Tables

**Table 1:** Descriptive statistics of body weight traits in Xianan cattle.

Trait	N	Mean	SD	Min	Max	CV (%)
BW (kg)	149	400.31	71.77	239	629.31	17.78

**Table 2:** Results of significant SNPs annotated using SnpEff database.

Chromosome	Position	Genes	Ref	Alt	Region	*p*_Value
9	54241895		C	T	intergenic_region	7.43 × 10^−9^
9	54239625		G	T	intergenic_region	1.88 × 10^−8^
9	54379055	MANEA	T	C	5_prime_UTR_variant	3.45 × 10^−8^
9	54326869	MANEA	T	C	intron_variant	3.63 × 10^−8^
9	54246066		T	C	intergenic_region	3.63 × 10^−8^
9	54246316		A	G	intergenic_region	6.22 × 10^−8^
9	54242338		C	T	intergenic_region	6.73 × 10^−8^
9	54393780		A	G	intergenic_region	7.05 × 10^−8^
5	32474126	HDAC7	A	G	5_prime_UTR_variant	1.41 × 10^−7^
9	54256092		C	T	intergenic_region	1.58 × 10^−7^
9	54261359		A	G	intergenic_region	2.55 × 10^−7^
5	32517886	RAPGEF3	G	A	upstream_gene_variant	2.54 × 10^−7^
1	121950885	PLSCR2	C	T	upstream_gene_variant	2.54 × 10^−7^
9	54339270	MANEA	T	A	intron_variant	2.50 × 10^−7^
9	54390222		G	A	intergenic_region	6.45 × 10^−7^
9	54266069		G	A	intergenic_region	6.41 × 10^−7^
9	54354464	MANEA	G	A	intron_variant	6.40 × 10^−7^
5	32476523	HDAC7	C	T	intron_variant	4.39 × 10^−7^
9	54315282	MANEA	T	C	intron_variant	6.29 × 10^−7^
9	54258450		A	C	intergenic_region	6.22 × 10^−7^

## Data Availability

Data is unavailable due to privacy.

## Appendix A

**Table A1:** Primers.

Primers	Sequence (5′ to 3′)	Base Number
MANEA-F	GATTCCCGGACCCTGCTAAA	20
MANEA-R	TGGTCTCAGCATTTTTAAACCCA	23
MGAT1-F	AAATGGAGTACTGGATGGGGG	21
MGAT1-R	GCAGCCATGCACCTTTCTTC	20
MGAT3-F	GCCGGAACCTCGTTGATGG	19
MGAT3-R	GCGTTTCATCTTCATCCCTGGC	22
FUT8-F	ATGGTGATCCTGCAGTGTGG	20
FUT8-R	CGTCTGACGTGGACTCCAAT	20
HK1-F	GAACGAATTTCCGCGTCCTG	20
HK1-R	TGTGGTCAAACAGCTCCTCC	20
GPI -F	GCTGGTGGACGTGGCTAAG	19
GPI -R	GCGTTTGATCGGTTCCGAAG	20
IL-6 -F	ACGAAAGAGAGCTCCATCTGC	21
IL-6 -R	AATGGAGTGAAGGCGCTTGT	20

**Table A2:** Significant SNPs detected by FST.

Chromosome	Position	Genes	Ref	Alt	Region	Log10 (*p*-Value)
9	54256092	LOC783932	C	T	intergenic region	0.411667
3	27516835		G	A	intergenic region	0.444595
3	27504770		C	G	intergenic region	0.479156
3	27516216		G	A	intergenic region	0.481131
9	54379055	MANEA	T	C	5_prime_UTR_variant	0.484033
15	26953056		A	G	intergenic region	0.484294
3	27509476		G	C	intergenic region	0.490252
19	49112923	TRNAE-UUC	A	G	upstream_gene_variant	0.494642
19	49110492		A	C	intron_variant	0.494642
19	49110226		A	G	intron_variant	0.494642
19	49108642		G	A	intron_variant	0.494642
28	4287845		T	G	intergenic_region	0.503047
15	26940189		T	C	intergenic_region	0.512241
15	26938941		T	C	intergenic_region	0.512241
6	111114247	CD38	C	T	intergenic_region	0.51408
3	89849741	TRNAW-CAA	G	A	intergenic_region	0.514114

**Table A3:** siRNA interference experiment.

Gene	Sample CT	18S rRNA	Ref Gene CT	2^−ΔΔCt^
NC	28.098	NC	18.536	1.000
NC	28.284	NC	18.541	——
NC	28.433	NC	18.604	——
MGAT1	18.393	MGAT1	17.960	599.219
MGAT1	18.739	MGAT1	18.118	——
MGAT1	18.782	MGAT1	18.387	——
NC	33.232	NC	17.408	1.000
NC	32.515	NC	17.425	——
NC	32.323	NC	17.489	——
MGAT3	31.995	MGAT3	17.605	1.501
MGAT3	31.767	MGAT3	17.456	——
MGAT3	32.784	MGAT3	17.472	——
NC	22.822	NC	17.339	1.000
NC	22.968	NC	17.620	——
NC	22.959	NC	17.352	——
FUT8	23.991	FUT8	18.130	0.784
FUT8	24.026	FUT8	18.093	——
FUT8	24.039	FUT8	18.341	——
NC	23.635	NC	18.247	1.000
NC	23.421	NC	18.060	——
NC	23.515	NC	18.122	——
HK1	26.633	HK1	20.305	0.521
HK1	26.744	HK1	20.182	——
HK1	26.638	HK1	20.525	——
NC	21.382	NC	17.195	1.000
NC	21.547	NC	17.057	——
NC	21.505	NC	17.517	——
GPI	22.554	GPI	18.195	0.836
GPI	22.661	GPI	18.063	——
GPI	22.690	GPI	18.241	——
NC	25.159	NC	17.876	1.000
NC	25.569	NC	17.501	——
NC	22.632	NC	17.525	——
IL-6	25.789	IL-6	18.575	1.275
IL-6	25.779	IL-6	18.640	——
IL-6	25.916	IL-6	18.430	——
